# Removal of Methylene Blue by Crosslinked Egg White Protein/Graphene Oxide Bionanocomposite Aerogels

**DOI:** 10.3390/nano12152659

**Published:** 2022-08-03

**Authors:** Yonghui Jin, Qiuju Du, Yanhui Li, Yang Zhang, Bing Chen, Mingzhen Wang, Kewei Chen, Yaohui Sun, Shiyong Zhao, Zhenyu Jing

**Affiliations:** 1 State Key Laboratory of Bio-Polysaccharide Fiber Forming, Eco-Textile, Qingdao University, 308 Ningxia Road, Qingdao 266071, China; jinyonghui1109@163.com (Y.J.); duqiuju@qdu.edu.cn (Q.D.); zy841876654lxm@outlook.com (Y.Z.); 2020020426@qdu.edu.cn (B.C.); wmz8805618@163.com (M.W.); ckw980518@163.com (K.C.); syh1866038168@163.com (Y.S.); zhao17862686144@163.com (S.Z.); jing1377959247@163.com (Z.J.); 2 College of Mechanical and Electrical Engineering, Qingdao University, 308 Ningxia Road, Qingdao 266071, China

**Keywords:** graphene oxide, egg white protein, aerogel, adsorption, methylene blue

## Abstract

Egg white protein is a non-toxic and biodegradable biopolymer that forms a gel easily via simple thermal denaturation treatment. A novel aerogel on the basis of egg white protein crosslinked with graphene oxide was prepared via a facile freeze-drying method. The structure and physicochemical characteristics of the aerogels were characterized by scanning electron microscopy (SEM), Fourier transform infrared (FTIR) spectroscopy, thermogravimetric analysis (TGA) and Brunauer–Emmett–Teller (BET) analysis. The adsorption properties of the aerogels were investigated by studying the influencing factors such as the solution pH, dose, temperature and contact time. The adsorption capacity of methylene blue onto the aerogels was tested, whose maximum adsorption capacity, calculated by the Langmuir isotherm equation, reached 91.7 mg/g. Adsorption kinetics studies showed that the adsorption followed the pseudo-second-order kinetic model. Thermodynamic data implied that methylene blue adsorbed by the aerogels was an exothermic and spontaneous process.

## 1. Introduction

Dyes as an important part of industrial production are applied to a variety of applications such as paper-making, cosmetic, textile, leather and printing. Many dyes contain quite complex molecular structures of aromatic compounds that are stable and non-degradable under heat or light, even in the presence of oxidants [1], so the discharge of water containing dyes into water body will pose a serious risk to humans as well as other creatures [2]. Therefore, the effective elimination of various dyes in wastewater is an important issue to solve the environmental pollution.

Methylene blue (MB) is a widely applied pigment in the industrial production. The dyes can enter the blood through the respiratory system, skin, and hair follicles, especially ingested through drinking water. MB will stimulate the gastrointestinal, nausea, vomiting, and diarrhea, even causing severe aplastic anemia or leukemia [3,4]. Therefore, it is essential to reduce aqueous methylene blue to acceptable levels prior to release into the environment, to adapt to increasingly standardized sewage discharge guidelines and strengthen the protection of natural ecology. Various physico-chemical processes such as photocatalytic degradation [5], ion exchange [6], biological treatment [7], electrochemical oxidation [8], flocculation-coagulation [9], and adsorption [10] have been contributing to sewage purification [11]. Due to the higher efficiency of the adsorption method, with the quite low application cost and the simpler operation, adsorption has become an effective method to remove low concentration dyes [12,13]. Therefore, it is urgently necessary and important to find more efficient adsorbents in the field of wastewater purification.

Graphene, an optimal two-dimensional nanomaterial with periodic honeycomb lattice structure, shows unique chemical and physical properties [14], thereby allowing it to be applied in a wide range of applications and various fields in fine detail, such as super capacitors [15,16], drug transport [17], sensors [18], batteries [19,20] and solar cells [21,22]. Superior to graphene in thermal properties, graphene oxide (GO) is a derivative of graphene with relatively prominent mechanical strength, large specific surface area, oxygen-containing molecular functional groups and rich hydrophilic groups [23]. With these characteristics, GO has become excellent absorbent to dyes and metal ions in recent years. However, its microscopic structure is quite small, and it cannot be completely separated from water via conventional methods after adsorption in sewage; moreover, its toxicity uncertainty to organisms prevents it from being used as adsorbent in practical environmental applications.

More recently, studies have been focused on searching for suitable carrier to prepare various GO nanocomposites. Polyacrylamide [24], cellulose [23], agar [25], xanthan gum [26], polyvinyl alcohol [27], chitosan [28], polystyrene [29], etc., have been screened to prepare GO macro composites, which separated the absorbents from the aqueous solution easily, reducing the potential risks of micro-sized GO to aquatic organisms. Egg white protein, a biomaterial, is a non-toxic and biodegradable biopolymer. It is not only rich in functional groups conducive to adsorption of amino (–NH_2_), hydroxyl (–OH) and carboxyl (–COOH) [30], but can also easily be coagulated to form a gel after thermal denaturation [31]. In this study, GO was dispersed into egg white protein solution in different weight ratios to fabricate egg white protein/graphene oxide (PGO) 3D aerogels. The important parameters affecting the adsorption of dye pollutants on PGO 3D aerogel, such as initial dye concentration, pH, dose, contact time and temperature were investigated.

## 2. Materials and Methods

### 2.1. Materials

Egg white protein was separated from eggs. Expandable graphite was purchased from Hengli Graphite Company (Qingdao, China). Methylene blue (C_16_H_18_ClN_3_S·3H_2_O) with a purity greater than 99% was purchased from China Sinopagic Chemical Reagents Co., Ltd. (Beijing, China). Potassium permanganate (KMnO_4_, 99.5%) and sodium nitrate (NaNO_3_, 99%) were purchased from Shanghai Jinlu Chemical Co., Ltd. (Shanghai, China). Sulfuric acid (H_2_SO_4_, 98%), hydrochloric acid (HCl) and hydrogen peroxide (H_2_O_2_) were purchased from Sinopharm Chemical Reagent Co., Ltd. (Beijing, China), with all reagents of analytical grade.

### 2.2. Preparation of GO

GO was prepared from expandable graphite via the improved Hummer method [32]. In the ice bath, H_2_SO_4_ (230 mL) was taken, NaNO_3_ (5 g) and KMnO_4_ (30 g) were added and completely immersed, and then the expansible graphite (5 g) was dispersed uniformly through continuous agitation. Subsequently, the temperature was set to 273 K, keeping the mixture at the temperature for 24 h. Afterwards, the mixture was stirred quickly until smooth and diluted slowly with deionized water at 308 K for 30 min. Whereafter, the reaction continued for 15 min at 371 K. The suspension changed from black to yellow by adding H_2_O_2_ (30%) to the mixed suspension slowly. Finally, the mixture was washed with HCl (5%) and centrifuged and purified with deionized water repeatedly to obtain GO.

### 2.3. Preparation of PGO Aerogels

As the primary method of preparing aerogel, the freeze-drying method is employed to prepare PGO aerogel. The protein content of egg white measured by freeze-drying method was 12%. First, the temperature was set to 293 K, 20 mL egg white was added to each beaker of 20 mL deionized water, whisking with a magnetic mixer for 1 h. Then, the prepared GO was added to the protein solution in proportions of 0, 10, 30, 50, 70 to 90 wt% in each beaker, stirring fully with a magnetic stirrer to obtain a uniform mixture. The mixture was placed in a Bain Marie at 373 K for 30 min until stable PGO hydrogel was obtained. Finally, the PGO aerogel was prepared through freeze-drying mechanism and freeze-dried for 48 h under vacuum (less than 20 Pa, −50 °C). PGO aerogels with various GO contents ranging from 0, 10, 30, 50, 70 to 90 wt% were labeled as PGO-0, PGO-10, PGO-30, PGO-50, PGO-70, and PGO-90, respectively.

### 2.4. Characterization of the Aerogels

The surface of PGO aerogel with various proportions was characterized by SEM (JSM 6700 F). The functional groups of PGO aerogel in different proportions were recorded employing FTIR spectrometer (Nicolet 5700), with the wave number set in the range of 4000~500 cm^−1^. The FTIR adopts ATR mode and diamond crystal. During TGA analysis which was carried out in a high-purity nitrogen atmosphere, the heating rate of the sample was 10 °C/min, and the curve did not change significantly in the range from 1073 K to 1273 K, so 1073 K was selected as the final temperature. The thermal stability was investigated at the temperature ranging from 303 to 1073 K on a thermogravimetric analyzer (METTLER TGA2, Columbus, OH, USA). The BET surface area of the adsorbent from the N_2_ adsorption isotherm at 77 K was measured by the Brunuer–Emmet–Teller equation (Quantachrome Autosorb-IQ-MP/XR, Boynton Beach, FL, USA). The specific surface area was measured by employing BET Micromeritics (Norcross, GA, USA). The machine model was ASAP.

### 2.5. Batch Adsorption Experiments

First, 1000 mg/L MB solution was prepared. In the adsorption experiment, samples were selected as cube-shaped integral aerogel adsorbents, each weighing 10 mg. Subsequently, the sample was added to a conical flask containing MB solution. A 50 mL conical flask was put into the water bath oscillator to oscillate according to the experimental variables. In the experiment, the sample was completely immersed in MB solution without collapse. After the adsorption experiment reached equilibrium, the concentration of different variables of MB solution was employing by using UV/Visible spectrophotometer (TU-1810). The adsorption capacity of MB to adsorbent can be calculated by the following formula:

(1) $$q_{e}=\frac{C_{0}-C_{e}}{m}V$$

where *C_e_* is the equilibrium concentration of MB solution (mg/L), *C*_0_ is the initial concentration of MB solution (mg/L); *m* is the weight of the adsorbent used (g) and *V* is the volume of the working solution (L).

In order to probe into the influence of initial pH value of solution on adsorption performance, 10 mg sample was added into 20 mL MB solution of 30 mg/L, and NaOH and HCl was added to adjust the initial pH of the solution from 2 to 10.

To investigate the effect of adsorbent dose on adsorption performance, a variety of mass samples (5–30 mg) were added into 20 mL MB solution with a concentration of 50 mg/L.

Aiming at studying the effect of different temperatures on the adsorption of MB solution, 10 mg samples were added to 20 mL solution, and the concentration of MB solution ranging from 5 to 50 mg/L, at 293, 303 and 313 K, respectively. Furthermore, the effect of exposure time was investigated by exposing 10 mg samples to 40 mL solutions with 15 and 20 mg/L MB. After the experiment setting time, the concentration of MB in the solution after adsorption was determined. At the current adsorption capacity, the adsorbent is calculated by the following formula:

(2) $$q_{t}=\frac{C_{0}-C_{t}}{m}V$$

where *C_t_* (mg/L) is the concentration of MB of the solution at time.

## 3. Results and Discussion

### 3.1. Characterization of the Samples

**Figure 1:**
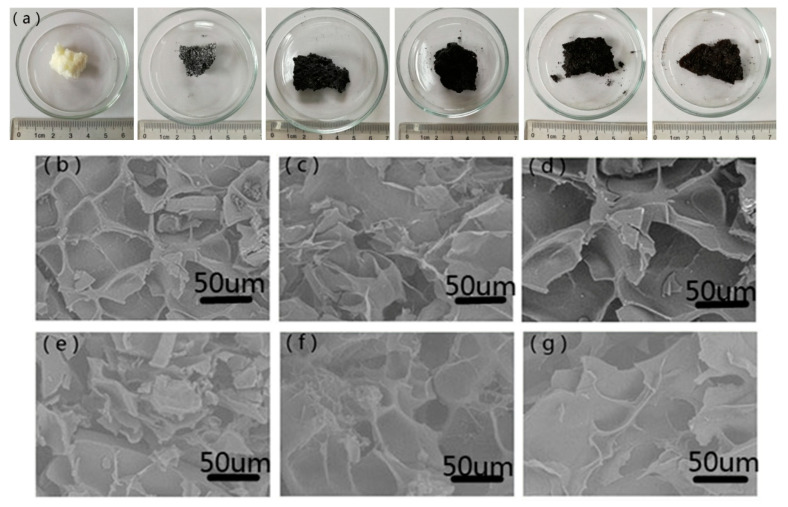
(**a**) The optical photographs of PGO aerogels, SEM images of PGO aerogels: (**b**) PGO-0, (**c**) PGO-10, (**d**) PGO-30, (**e**) PGO-50, (**f**) PGO-70, and (**g**) PGO-90.

Figure 1 shows the optical photographs and SEM images of PGO aerogels. All the samples prepared through freeze drying demonstrate 3D structures (Figure 1a). PGO aerogels show good formability at GO weight percent ranging from 0 to 30%, whereas the formability becomes weak when the percent exceeds 50%. Figure 1b–g show that all of PGO aerogels present porous structure. In terms of the process of freeze drying, the sample is first frozen into a solid as the water turns into ice which then sublimates directly to remove water without changing its solid state at low and low temperatures. Due to sublimated water molecules, the first position occupied by ice becomes porous.

**Figure 2:**
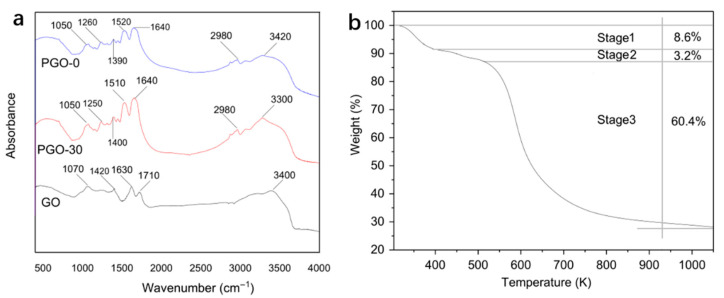
(**a**) FT-IR spectra of PGO-0, GO, PGO-30, and (**b**) TGA curves of PGO-30.

The FTIR spectra of PGO-0, GO and PGO-30 aerogels were measured, as shown in Figure 2a. There are peak values at 3420, 2980, 1640, 1520, 1260 and 1050 cm^−1^ in the FTIR spectrum of PGO-0, corresponding to O–H, C–H, C=C, N–H, C=C, and C–O flexion and extension vibration, respectively [33]. The FTIR spectra of GO contain peaks of 3400, 1710, 1630, 1420 and 1070 cm^−1^, corresponding to flexion and extension vibration of O–H, C=O, C=C and C–O, respectively [34]. All peaks in FT-IR spectra of PGO-0 and GO appear in the FT-IR spectrum of PGO-30 aerogels, indicating that the egg white protein combine well with GO to form a novel composite.

The TGA curve of PGO aerogels (PGO-30) is shown in Figure 2b. During TGA analysis which was carried out in a high-purity nitrogen atmosphere, the heating rate of the sample was 10 °C/min, and the curve did not change significantly in the range from 1073 K to 1273 K, so 1073 K was selected as the final temperature. The results showed that the pyrolysis process of PGO aerogel exhibited three obvious degradation stages. To be specific, the dehydration and desiccation process in the first stage resulted in an 8.6 percent weight loss from 293 K to 357 K. In the second stage, oxygen-containing functional groups gradually disappeared in GO gradually, causing a little weight loss (3.2%) from 357 K to 453 K. In the final stage, a large amount of pyrolysis of the egg white protein and oxidation of GO brought in a dramatic increase in weight loss (60.4%) from 453 K to 1073 K [35].

**Figure 3:**
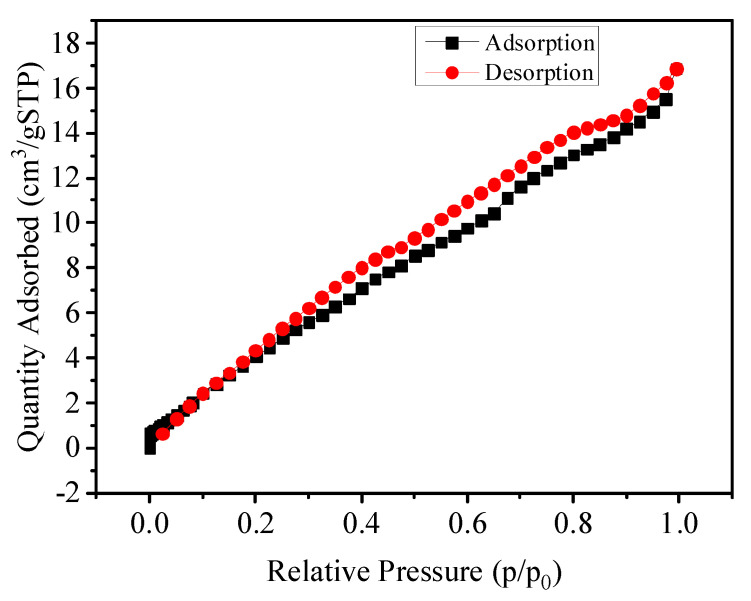
Nitrogen adsorption–desorption isotherm of PGO aerogel.

Figure 3 shows the nitrogen adsorption–desorption isotherms of the sample. The specific surface area was measured by employing BET Micromeritics (USA). The machine model was ASAP. The specific surface area of PGO-30 aerogels was 43 m^2^/g, respectively.

### 3.2. Adsorption of MB

**Figure 4:**
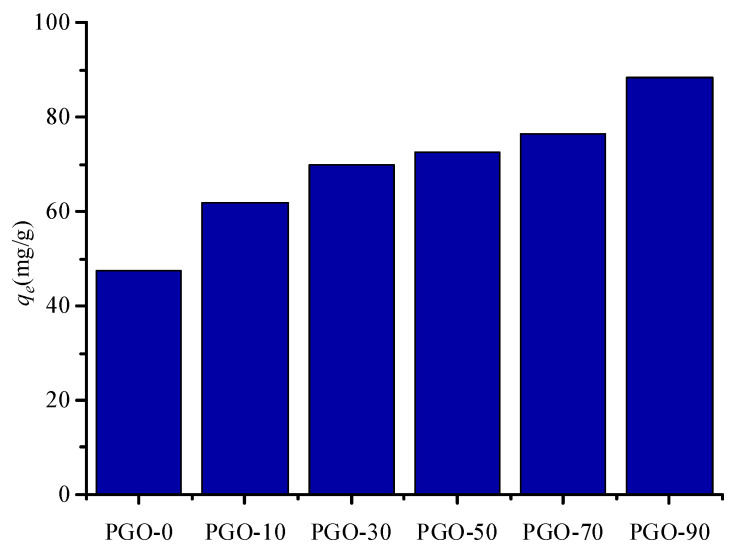
Adsorption capacity of PGO aerogels with different GO contents.

To investigate the disparate effects of GO content in aerogel on MB adsorption, the temperature was set at 293 K, and 10 mg of different adsorbents were added into 20 mL MB solution with an initial concentration of 50 mg/L. The adsorption capacity of pure egg white protein aerogel (PGO-0), which is only 47.6 mg/g, is shown in Figure 4. As 10 wt% GO mixed with the egg white protein, the adsorption capacity increased significantly, reaching 61.8 mg/g, which increased it continually with the increase of GO content, reaching 88.4 mg/g at GO content of 90 wt%. Although the higher GO content in aerogel could contribute to improving the adsorption of MB, the mechanical property of the aerogels with higher GO content become weak. After the adsorption, the aerogels were easily dispersed into the solution and difficult to be removed via conventional separation method, helping explain that PGO-30 was selected as the main experimental sample.

#### 3.2.1. Effect of Initial pH

**Figure 5:**
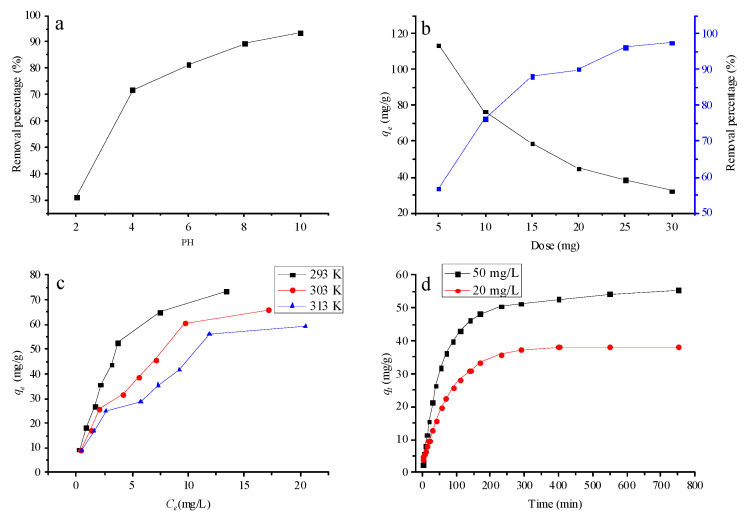
Effect of different factors on MB adsorbed by PGO-4 aerogel: (**a**) pH effect, (**b**) dose effect, (**c**) temperature effect, and (**d**) contact time effect.

As a key parameter affecting the adsorption performance of dyes [36], the initial pH value in solution affects not only the properties of adsorbents, but also the surface charge of adsorbents. The influence of the initial pH value of the solution on the adsorption MB of PGO-30 aerogel is shown in Figure 5a. Obviously, the removal percentage raises from increased 31.1 to 93.6% as the initial pH increased from 2.0 to 10.0. The lower removal rate at lower initial pH may be attributed to the competition between hydrogen ions and MB molecules at available binding sites [37]. With an increase in initial pH, the carboxyl group and part of the hydroxyl group of PGO aerogel may be deprotonated to form –COO– and part of –O– groups. The mutual electrostatic attraction between the MB cation and the negatively charged surface of PGO aerogel may help explain the increase in adsorption capacity [38].

#### 3.2.2. Effect of Adsorbent Dose

The effects of absorbent dose on removal percentage and adsorption capacity are shown in Figure 5b. With the increase of adsorbent dose, the removal rate of MB on PGO aerogel increased from 58.5 to 97.2%, which was attributed to a large increase in numerous adsorption sites and a large increase in surface area [39]. Nevertheless, the adsorption capacity gradually decreased with the increasing adsorption dose. It may be owing to the reduced utilization rate of the adsorbents, and only parts of active sites on PGO aerogels were employed to adsorb MB molecules [40,41].

#### 3.2.3. Effect of Temperature

The influence of temperature on the equilibrium adsorption capacity at 293 K, 303 K and 313 K is shown in Figure 5c. With the temperature rising from 293 K to 313 K, the maximum adsorption capacity of PGO aerogels decreased from 73.3 mg/g to 59.0 mg/g. The experimental results indicate that the adsorption process of MB on PGO aerogel is exothermic.

#### 3.2.4. Effect of Contact Time

Figure 5d shows the influence of contact time of PGO aerogel on adsorption at two different initial concentrations. It can be observed that the adsorption rate was quite fast within the initial 100 min, which can be attributed to the exposed surface area of MB molecule PGO aerogel and the rapid contact with the active site. Thereafter, the adsorption rate increased gradually until equilibrium [42]. The long-distance diffusion of dye molecules into the inner pores of the adsorbent particles could help explain the decrease in the final adsorption rate. Moreover, Figure 5d demonstrates that the adsorption capacity and equilibrium adsorption time were significantly affected by initial MB concentration. As the initial concentration of MB increased from 15 to 20 mg/L, they increased from 38.2 mg/g to 55.4 mg/g and from 400 min to 750 min, respectively.

### 3.3. Adsorption Isotherms

**Figure 6:**
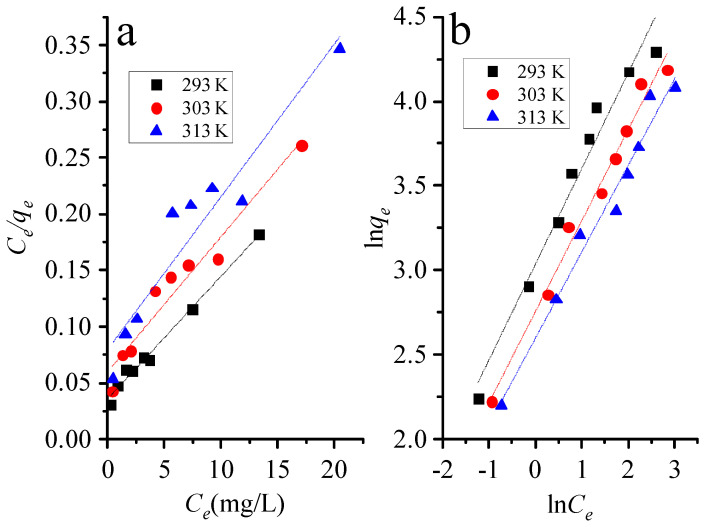
(**a**) Langmuir and (**b**) Freundlich models of adsorption MB on PGO aerogels at different temperatures.

The adsorption properties of adsorbents were characterized by adsorption isotherm model and the relationship between adsorbents and adsorbents was described. The Langmuir model assumed that adsorption occurs on a uniform and noninteracting plane. The Langmuir isotherm equation follows:

(3) $$\frac{1}{q_{e}}=\frac{1}{q_{\max}}+\frac{1}{q_{\max}k_{L}C_{e}}$$

where *k_L_* (L/g) is Langmuir constant, *C_e_* (mg/L) is the equilibrium concentration, *q*_e_ (mg/g) is the amount adsorbed at equilibrium, and *q*_max_ (mg/g) represents the maximum adsorption capacity. *C_e_* and *C_e_/q_e_* can be obtained from experimental data, *q_max_* as well as *k_L_* are calculated by linear fitting. Figure 6a shows the Langmuir isotherms at 293, 303 and 313 K, and the parameters are shown in Table 1.

The dimensionless equilibrium parameter *R_L_* can be expressed by Langmuir isotherms:

(4) $$R_{L}=\frac{1}{1+k_{L}C_{0}}$$

where *R_L_* value indicates whether Langmuir isotherms are irreversible (*R_L_* = 0), favorable (0 < *R_L_* <1), linear (*R_L_* = 1) or unfavorable (*R_L_* > 1). As can be seen from Table 1, since the *R_L_* value is between 0 and 1, PGO aerogel is a favorable adsorbent for removing MB.

The Freundlich model shows that adsorption is carried out at molecular level through adsorption sites and functional groups. The Freundlich isothermal equation follows:

(5) $$Lnq_{e}=Lnk_{F}+\frac{1}{n}Lnc_{e}$$

where *k_F_* (L/g) is the Freundlich constant related to adsorption capacity, and *n* is the Freundlich constant related to adsorption strength. *Lnq_e_* and *LnC_e_* can be obtained from experimental data, *Lnk_F_* as well as 1/*n* are calculated by linear fitting. Figure 6b shows the Freundlich isotherm plots at 293 K, 303 K and 313 K, whose parameters are listed in Table 1. The determination coefficient R^2^ of the Freundlich isotherm was greater than that of the Langmuir isotherm, so the Freundlich adsorption model was more appropriate to describe the adsorption data. The adsorption has a favorable removal condition because the value of 1/*n* obtained from the Freundlich model equation is less than 1.

### 3.4. Kinetic Studies

Adsorption kinetics serves as an important index for analyzing the experimental process of adsorption in evaluating the performance of adsorbents. The adsorption data were fitted based on the model of intra-particle diffusion, pseudo-first-order model and pseudo-second-order model.

**Figure 7:**
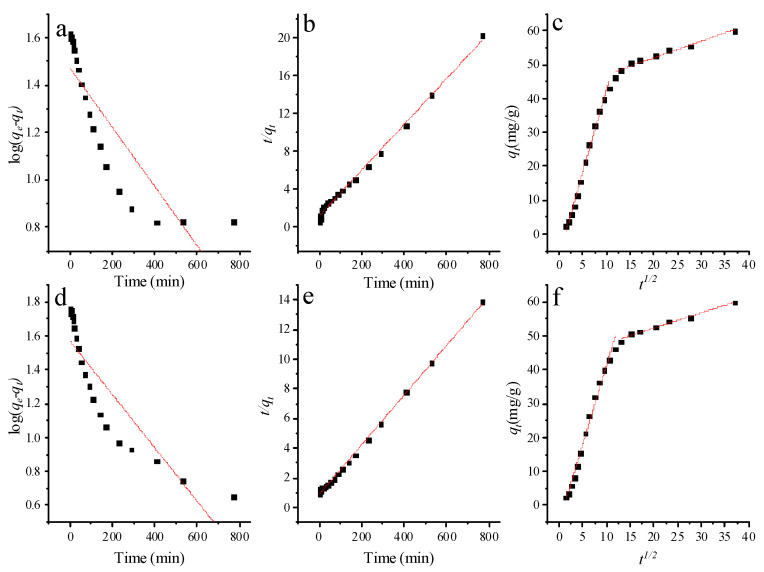
Adsorption kinetics of MB on PGO aerogel: (**a**) and (**d**) pseudo first-order model, (**b**) and (**e**) pseudo second-order model, and (**c**) and (**f**) intra-particle diffusion model.

The pseudo-first-order model equation is expressed by the following formula [43]:

(6) $$\log(q_{e}-q_{t})=\log q_{e}-\frac{k_{1}}{2.303}t$$

where *k*_1_ (min^−1^) is the rate constant of the pseudo-first-order model. As shown in Table 2, parameters *q_e_* and *k*_1_ can be calculated by fitting the intercept and slope of the graph with experimental data (Figure 7a,d), respectively. The values of R^2^ were 0.784 (*C*_0_ = 20 mg/L) and 0.733 (*C*_0_ = 15 mg/L), respectively. The lower values of R^2^ show that the adsorption of PGO aerogel does not conform to the pseudo-first-order kinetic model.

The pseudo-second-order model equation is expressed by the following formula [44]:

(7) $$\frac{t}{q_{t}}=\frac{1}{k_{2}q_{e}{}^{2}}+\frac{t}{q_{e}}$$

where *k*_2_ (g/mg min) represents the rate constant of the pseudo-second-order model.

As shown in Table 2, the values of *k*_2_ and *q_e_* can be obtained by fitting the experimental slope and intercept data of the graph (Figure 7b,e). The comparison between the two models indicates obviously that the coefficient values of the pseudo-first-order dynamics model are not as consistent as the determined coefficient values of the pseudo-second-order dynamics model. Therefore, the kinetic adsorption data of PGO aerogel adsorption MB is more consistent with the pseudo-second-order kinetic model.

Compared with other models, the intra-particle diffusion model is more suitable to show the kinetics of the diffusion process of intra-particle adsorption, and the equation of the intra-particle diffusion model is shown as follows [45]:

(8) $$q_{t}=k_{id}t^{1/2}+C_{i}$$

where *k_id_* (mg/g min^1/2^) is the diffusion rate constant within the particle, *t*^1/2^ and *C* represent the square root of time and boundary layer have a great influence on molecular diffusion, respectively. The slope and intercept of the fitting graph of the experimental data (Figure 7c,f) were calculated to obtain the rate constants *k_id_* and *C_i_* (Table 2).

Obviously, the value of *C* is not equal to zero, with the fitting line being non-linear. The intra-particle diffusion can be classified into two stages by the fitting the graph. The first stage of adsorption is instantaneous adsorption or surface adsorption, where the high removal rate of MB is attributed to the large specific surface area and large active adsorption site. The second stage is a slow adsorption process, suggesting that MB molecules are absorbed into the inner pores of PGO aerogel controlled by the diffusion rate.

### 3.5. Adsorption Thermodynamic

The adsorption thermodynamic parameters of MB on PGO aerogel were measured at different temperatures in order to study the influence on the adsorption process. Entropy change (Δ*S*) and enthalpy change (Δ*H*) are calculated by van’t Hoff equation [46,47]. The formula is as follows:

(9) $$Ln\left(\frac{q_{e}}{C_{e}}\right)=-\frac{\Delta H}{RT}+\frac{\Delta S}{R}$$

The Gibbs free energy change (Δ*G*) is calculated as follows:

(10) ΔG=ΔH−TΔS

where *T* is the temperature in Kelvin (K), *q_e_* (mg/g) and *R* are the adsorption capacity and the universal gas constant (8.314 J/mol∙K), respectively. Using the slope of the linear fitting graph and the intercept of the linear line, the values of −Δ*H/R* and Δ*S/R* are further calculated. Calculate the values of Δ*G*, Δ*H*, and Δ*S* as shown in Table 3.

Since the negative enthalpy change (Δ*H* = −24.6 KJ/mol) means that the absorbed energy is less than the released energy, adsorption is proved to be an exothermic process. With the rising temperature, the adsorption capacity decreases gradually, thus supporting the conclusion of temperature effect. The change of negative entropy (Δ*S* = −69.90 J/mol∙K) indicates that the adsorption at the solid-solute interface increases randomly during the adsorption process. Negative Δ*G* indicates that MB adsorption on PGO aerogel is a spontaneous reaction.

In Table 4, the maximum removal rate of MB by different adsorbents is compared. Wu et al. studied the adsorption performance of Composite response hydrogels of Huangshui polysaccharide, polyvinyl alcohol and carboxymethyl sodium cellulose for MB, and obtained paramagnetic porous hydrogels through physical cross-linking, with the maximum adsorption capacity of MB being 71.07 mg/g [48]. Noori, M. et al. prepared Clinoptilolite/Fe_3_O_4_ (Clin/Fe_3_O_4_) nanocomposite powders and Alginate/Clinoptilolite/Fe_3_O_4_ (Alg/Clin/Fe_3_O_4_) nanocomposite beads to remove MB, and investigated the effects of contact time, temperature, pH, amount of adsorbent and initial concentration on adsorption performance. The maximum adsorption capacity was 45.662 mg/g and 12.484 mg/g, respectively [49]. Mekuria, D. et al. selected barley (Hordeum Vulgare) bran (BB) and Enset (Ensete ventricular middle costal leaf, EVML) as adsorbents to study the removal of MB from wastewater. The adsorbent has good adsorption performance in a wide range of Ph values. The maximum adsorption capacity for MB is 63.2 mg/g (BB) and 35.5 mg/g (EVML), respectively [50]. Wu et al. studied the adsorption of copper and methylene blue (MB) in aqueous solution using natural wheat straw as adsorbent. There is no significant difference in adsorption capacity for MB when the pH range is 4.0~10.0. The adsorption of MB accords with the Redlich–Peterson model. The maximum adsorption capacity of MB is 60.66 mg/g [51]. The effects of pH, temperature, contact time, amount of adsorbent and initial concentration of dye on the adsorption properties have been investigated in all the above articles. The thermodynamic parameter analysis shows that the parameter Δ G of all samples is negative, indicating that the process is spontaneous. By comparison, it is similar to the experimental process in this paper. The egg white protein/GO composite aerogel whose maximum adsorption capacity to MB is 91.7 mg/g has a broad application prospect in MB removal.

## 4. Conclusions

In summary, novel PGO composite aerogels were prepared by egg white protein cross-linking with GO. The potential application of PGO aerogel for dye removal in wastewater was described. The adsorption kinetics of MB on PGO aerogel follows the pseudo-second-order kinetic model. The adsorption isotherm data fit well with Freundlich model. According to thermodynamic parameters, the adsorption of MB by PGO aerogel is a spontaneous exothermic process. The results show that PGO aerogel can be used as an excellent adsorbent for MB removal from wastewater.

## Figures and Tables

**Table 1:** Isothermal constants for MB adsorption on PGO aerogels.

Temp (K)	Langmuir				Freundlich		
	*q*_max_ (mg/g)	*k_L_* (L/mg)	R^2^	*R_L_*	*k_F_* (L/mg)	1/*n*	R^2^
293	91.7	3.26	0.987	0.0579	20.8	0.57	0.962
303	83.3	4.98	0.940	0.0386	15.7	0.54	0.982
313	73.8	5.87	0.900	0.0330	13.4	0.51	0.972

**Table 2:** The kinetic constant of adsorption of MB on PGO aerogels.

*C*_0_ (mg/L)		20	50
Pseudo-first-order model	*k*_1_ (min^−1^)	2.88 × 10^−3^	3.62 × 10^−3^
	*q_e_* (mg/g)	29.6	37.1
	R^2^	0.733	0.784
Pseudo-second-order model	*k*_2_ (g/mg∙min)	4.79 × 10^−4^	3.20 × 10^−4^
	*q_e_* (mg/g)	41.6	59.8
	R^2^	0.995	0.998
Intraparticle diffusion model	*k_id_*_1_	4.87	4.64
	*C*_1_	−6.02	−5.05
	R_1_^2^	0.991	0.986
	*k_id_*_2_	0.493	0.449
	*C*_2_	42.1	43.3
	R_2_^2^	0.944	0.976

**Table 3:** Thermodynamic parameters at different temperatures.

T/K	Δ*G* (kJ/mol)	Δ*H* (kJ/mol)	Δ*S* (J/mol∙K)
293	−4.13	−24.6	−69.9
303	−3.43		
313	−2.73		

**Table 4:** Comparison of maximum removal capacities of different adsorbent materials for MB.

Adsorbent Material	Removal Capacity (mg·g^−1^)	Ref.
Hydrogels loaded with Huangshui polysaccharides, polyvinyl alcohol, and sodium carboxyl methyl cellulose	71.07	[48]
Clinoptilolite/Fe_3_O_4_(Clin/Fe_3_O_4_) nanocomposite powders	45.662	[49]
Alginate/Clinoptilolite/Fe_3_O_4_ (Alg/Clin/Fe_3_O_4_) nanocomposite beads	12.484	[49]
Barley Bran	63.2	[50]
Enset Midrib Leaf	35.5	[50]
Wheat Straw	60.66	[51]
Egg White Protein/Graphene Oxide Bionanocomposite Aerogels	91.7	This study

## Data Availability

The data presented in this study are available on request from the corresponding authors.
